# How Much Rugby is Too Much? A Seven-Season Prospective Cohort Study of Match Exposure and Injury Risk in Professional Rugby Union Players

**DOI:** 10.1007/s40279-017-0721-3

**Published:** 2017-03-30

**Authors:** Sean Williams, Grant Trewartha, Simon P. T. Kemp, John H. M. Brooks, Colin W. Fuller, Aileen E. Taylor, Matthew J. Cross, Gavin Shaddick, Keith A. Stokes

**Affiliations:** 1 Department for Health, University of Bath, Bath, BA2 7AY UK; 2 Rugby Football Union, Twickenham, UK; 3 The Population Health Research Institute, St. George’s University of London, London, UK; 4 Colin Fuller Consultancy, Sutton Bonington, UK; 5 Karabati Limited, Nottingham, UK; 6 Department of Mathematical Sciences, University of Bath, Bath, UK

## Abstract

**Introduction:**

Numerous studies have documented the incidence and nature of injuries in professional rugby union, but few have identified specific risk factors for injury in this population using appropriate statistical methods. In particular, little is known about the role of previous short-term or longer-term match exposures in current injury risk in this setting.

**Objectives:**

Our objective was to investigate the influence that match exposure has upon injury risk in rugby union.

**Method:**

We conducted a seven-season (2006/7–2012/13) prospective cohort study of time-loss injuries in 1253 English premiership professional players. Players’ 12-month match exposure (number of matches a player was involved in for ≥20 min in the preceding 12 months) and 1-month match exposure (number of full-game equivalent [FGE] matches in preceding 30 days) were assessed as risk factors for injury using a nested frailty model and magnitude-based inferences.

**Results:**

The 12-month match exposure was associated with injury risk in a non-linear fashion; players who had been involved in fewer than ≈15 or more than ≈35 matches over the preceding 12-month period were more susceptible to injury. Monthly match exposure was linearly associated with injury risk (hazard ratio [HR]: 1.14 per 2 standard deviation [3.2 FGE] increase, 90% confidence interval [CI] 1.08–1.20; likely harmful), although this effect was substantially attenuated for players in the upper quartile for 12-month match exposures (>28 matches).

**Conclusion:**

A player’s accumulated (12-month) and recent (1-month) match exposure substantially influences their current injury risk. Careful attention should be paid to planning the workloads and monitoring the responses of players involved in: (1) a high (>≈35) number of matches in the previous year, (2) a low (<≈15) number of matches in the previous year, and (3) a low-moderate number of matches in previous year but who have played intensively in the recent past. These findings make a major contribution to evidence-based policy decisions regarding match workload limits in professional rugby union.

**Electronic supplementary material:**

The online version of this article (doi:10.1007/s40279-017-0721-3) contains supplementary material, which is available to authorized users.

## Key Points

**Table:** 

Players who have been involved in a low (<15) or high (>35) number of matches over the previous 12 months are more susceptible to injury, so their workloads and responses to workloads should be carefully monitored and managed.
Involvement in 35 matches over a 12-month period should be considered as an upper limit for professional rugby union players.
Injury risk rises with increases in 1-month match exposures, particularly for those with low chronic (1-year match exposure) exposure to matches. Players returning from long absences from match play should do so in a graduated manner.

## Introduction

Injury incidence and the resulting absence from match play and training in elite rugby union are high in comparison with most team sports [1], and the incidence of injuries at the team level are negatively associated with team success [2]. The identification of risk factors for injury, especially those that are modifiable, is a key component in the development of effective injury-prevention strategies [3]. Whilst numerous studies have documented the incidence and nature of injuries in professional rugby union (for review, see Williams et al. [1]), few have identified specific risk factors for injury in this population using appropriate statistical methods. In particular, little is known about the effect of previous short-term or longer-term match exposures upon current injury risk in this setting.

The introduction of professional full-time training, advancements in sports science, and law changes in rugby union have resulted in marked changes in players’ physical characteristics [4] and match activities [5] over recent decades. The result of such changes (e.g., more frequent collisions [5] of greater magnitude) has engendered media attention regarding the potential long-term consequences of ‘excessive’ match exposure demands being placed on professional rugby union players [6, 7]. Whilst qualitative investigations have attributed factors such as limited recovery time in the off season and an ‘anti-rest culture’ as causes for burnout syndrome and increased injury risk in rugby union players [8, 9], these loading issues have not been examined quantitatively in this setting. Alongside these ‘cumulative’ match workload questions, there is also evidence to suggest that recent match workloads may be associated with injury risk in some elite sports settings. In professional soccer, for example, congested fixture periods have been shown to increase injury risk in the ensuing period [10, 11]. In addition, the interaction between acute (1-week) and chronic (4-week rolling average) training loads has been highlighted as an important predictor of injury [12]. However, the impact of both recent and accumulated match exposure upon injury risk in this setting is currently unclear. Such data have important implications relating to fixture scheduling (e.g., the scheduling of off-season and within-season breaks) and player match exposure limits in professional rugby union.

Although sports injury data often contain repeated events within individuals (e.g., multiple injuries and/or data across multiple seasons), few published studies have considered how these repeated measurements impact upon the statistical assumptions made in their analyses, leading to potentially spurious conclusions [13]. As several works highlight [14, 15], to progress injury prevention in sport there is a clear need to appropriately account for the multifactorial and dynamic nature of sports injuries. The frailty model has been identified as the most suitable statistical approach for analyzing recurrent sports injury data of this nature [16]. Specifically, the frailty model accommodates censored observations, highly skewed data, and time-varying covariates [17] whilst making fewer statistical assumptions than other survival models [16]. However, to our knowledge, the application of nested models to account for within-team correlations, in addition to within-player correlations, has yet to be undertaken in sports epidemiology settings. Accordingly, the aim of the present study was to assess the influence that recent and accumulated match exposures have upon injury risk for professional rugby union players through the application of a nested frailty model for recurrent events.

## Methods

A seven-season prospective cohort design was used to record all match and training injuries sustained by professional rugby union players in the English premiership. Data collected from the 12 league teams in each of the seven seasons between 2006/07 and 2012/13 were included in the analysis, giving rise to a total of 15 teams because of promotions and relegations during this period. All consenting players who were members of the first team squad were eligible for inclusion. Data pertaining to 1253 professional rugby union players were included in the analysis (mean ± standard deviation [SD] age = 26 ± 4 years; height = 186 ± 8 cm; mass = 102 ± 13 kg; number of previous time-loss injuries = 6 ± 6 injuries). The study was approved by the research ethics committee of the academic host institution where the project was based for each season, and written informed consent was obtained from each participant. All data were anonymized, and all procedures were performed in accordance with the Declaration of Helsinki [18].

The injury definition used in this study was ‘Any physical complaint sustained by a player during a first-team match or training session that prevented the player from taking a full part in all training activities typically planned for that day, and/or match play for more than 24 h from midnight at the end of the day the injury was sustained’ [19]. All injuries were recorded by medical personnel for each team using a modified Orchard Sports Injury Classification System (OSICS) [20] and standard injury report form. Individual match and group training exposure data (h) were reported weekly by each team.

Accumulated match exposure was calculated as the number of matches in which a player participated (for ≥20 min) during the preceding 12-month period (12-month match exposure). Match involvements of ≥20 min were used to allow meaningful substitute appearances to be captured. Moreover, involvements of <20 min are typically excluded in match analysis studies [21, 22]. Recent match exposure was calculated as a player’s full-game equivalent [FGE] match exposure (total match exposure in minutes divided by 80) in the preceding 30 days (1-month match exposure). These timeframes were selected to best inform prominent questions relating to fixture congestion and season structures in team sports [23]. Accumulated match exposure was calculated on the basis of number of match involvements (i.e., an integer value) because the loads associated with preparation for such involvements (e.g., travel, training, and performance analysis) are also likely to influence injury risk [23]. However, for 1-month match exposure, this approach produced limited variation in the predictor variable, so we used the number of FGEs (i.e., a continuous variable) instead. The interaction between these two variables was also investigated: the 12-month match exposure variable was parsed into quartiles and included as a multiplicative term with 1-month match exposure in the nested frailty model. Predictor variables were calculated at each injury or censored event time point. A nested frailty model was applied to the injury data to calculate adjusted hazard ratios (HRs) of injury risk with 90% confidence intervals (CIs) for the assessed risk factors. Injury risk related to the HR (i.e., the instantaneous risk of injury, given survival to time *t*) for both match and training time-loss injuries. The nested frailty model included two random effects to describe hierarchical grouping in the data (i.e., within-team and within-player correlations) [24]. The HRs were adjusted by controlling for players’ age, mass, height (as continuous variables), playing position (forward/back), and previous injury history (number of previous injuries in the dataset), and were offset for individual match exposure and team training exposure since the return from their previous injury (gap time). Players with 12-month match exposures of zero were excluded from the analysis. Models were fitted using the *Coxme* package [25] with R (version 3.2.4, R Foundation for Statistical Computing, Vienna, Austria). Modified Wald tests were used to determine whether the variance parameter from the frailty models was significantly different from zero [26].

We examined whether responses were non-linear, as recommended by Gabbett et al. [27], by including quadratic and cubic terms in the model. Otherwise, linear effects for continuous predictor variables were evaluated as the change in injury risk associated with a two SD increase in the predictor variable [28]. Magnitude-based inferences were used to provide an interpretation of the real-world relevance of the outcome, based directly on uncertainty in the true value of the outcome variable in relation to a smallest worthwhile effect [29]. Thresholds for beneficial and harmful effects were HRs of 0.90 and 1.11, respectively [30]. Effects were classified as unclear if the ±90% CIs crossed thresholds for both beneficial and harmful effects by >5%. Otherwise, the effect was clear and deemed to have the magnitude of the largest observed likelihood value: beneficial if associated with decreased injury risk, harmful if associated with increased injury risk, and trivial if associated with a non-substantial (below the smallest worthwhile change threshold) change in injury risk. This was qualified with a probabilistic term using the following scale: <0.5%, most unlikely; 0.5–5%, very unlikely; 5–25%, unlikely; 25–75%, possible; 75–95%, likely; 95–99.5%, very likely; and > 99.5%, most likely [31].

To evaluate the utility of the nested frailty model, the log likelihood (LL), Akaike information criterion (AIC) and Bayesian information criterion (BIC) values were used to assess and compare the goodness of fit of the nested model to other potential models: a Cox proportional hazards (Cox PH) model (i.e., a survival model without random effects [32]) and a shared frailty model that used a single random effect to describe within-player grouping only [33]. Smaller LL, AIC, and BIC values indicated a better fit to the observed data [34]. The *anova.coxme* function was used to compare the change in LL for each survival model, with significance accepted at an *α* level of *p* ≤ 0.10. A difference in AIC and BIC values of >2 was accepted as evidence of substantial differences [35].

## Results

A total of 6890 time-loss injuries (match: 5029; training: 1861) were recorded over the study period. The average incidence rate over the study period was 85.9 ± 9.0 per 1000 player h for match injuries and 2.8 ± 0.4 per 1000 h for training injuries. Of the included players, 78% incurred two or more time-loss injuries over the study period. Mean 12-month match exposures were 18.8 ± 9.6 matches (range 1–40) and mean 1-month match exposures were 1.7 ± 1.6 FGE matches (range 0–5).

**Fig. 1:**
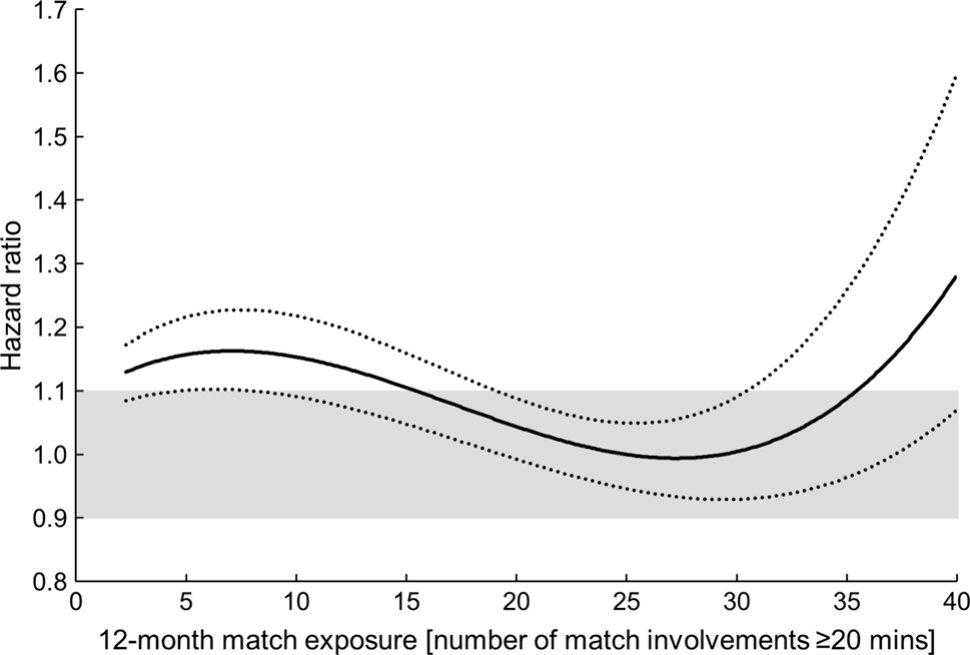
Non-linear association between injury risk and 12-month match exposure, with 90% confidence intervals. *Shaded area* represents thresholds for benefit (hazard ratio: 0.90) and harm (hazard ratio: 1.10)

**Fig. 2:**
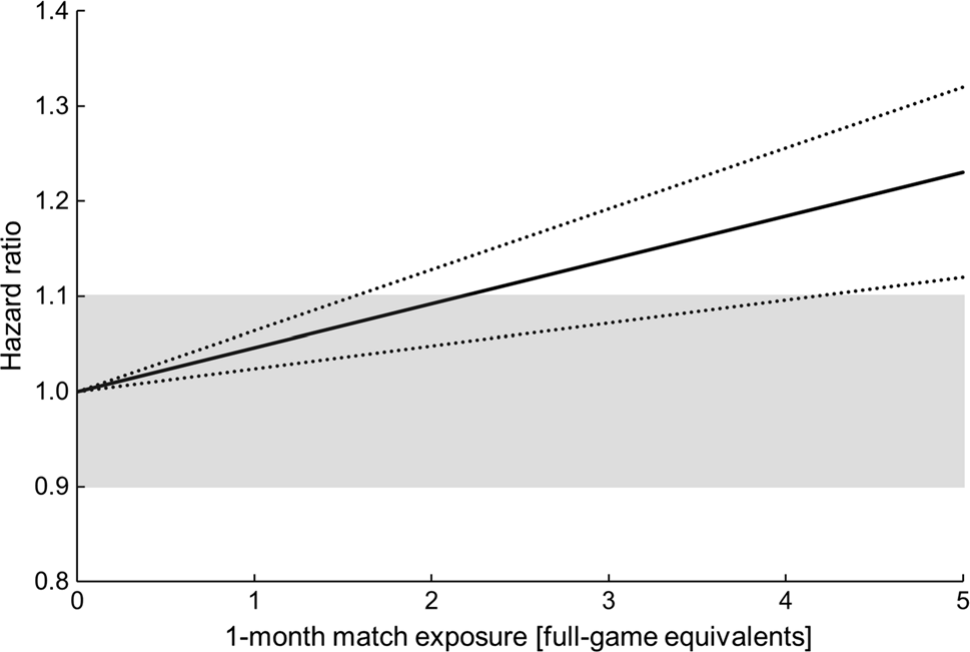
Linear association between injury risk and 1-month match exposure, with 90% confidence intervals. *Shaded area* represents thresholds for benefit (hazard ratio: 0.90) and harm (hazard ratio: 1.10)

Evidence of a non-linear relationship with injury risk was found for the 12-month match exposure variable, with a cubic function providing the best model fit (Fig. 1). A substantial increase in injury risk was evident for players who were involved in fewer than ≈15 or more than ≈35 matches over the preceding 12-month period. No evidence of a non-linear relationship was observed for the 1-month match exposure variable. A 2-SD increase in 1-month match exposure was associated with an HR of 1.14 (90% CI 1.08–1.20; likely harmful) (Fig. 2). There was evidence of an interaction effect between 12-month match exposures and 1-month match exposures (Table 2), with players in the highest quartile of 12-month match exposure (28–40 matches) having a likely beneficial reduction in HR compared with players on the lowest quartile (<12 matches). The effects associated with covariates (age, height, mass, positional group, and number of previous injuries) included in the nested frailty model are presented in Table 1; all effects were ‘trivial’, with the exception of ‘number of previous injuries’, for which a 2-SD increase (six injuries) was associated with an HR of 1.28 (90% CI 1.15–1.41; very likely harmful).

**Table 1:** Effects associated with covariates appearing in the nested frailty model

Covariate	HR (90% CI)	*p* value	Inference	% likelihood effect is beneficial |trivial| harmful
Age (2 SDs = 8 y)	1.03 (0.98–1.09)	0.32	Very likely trivial	0 |99| 1
Height (2 SDs = 15 cm)	1.05 (0.99–1.13)	0.19	Likely trivial	0 |93| 7
Mass (2 SDs = 26 kg)	1.02 (0.95–1.11)	0.62	Likely trivial	0 |95| 5
Positional group (reference = ‘Backs’)	0.91 (0.85–0.98)	0.04	Possibly trivial	39 |61| 0
Number of previous injuries (2 SDs = six injuries)	1.28 (1.15–1.41)	0.0001	Very likely harmful	0 |1| 99

*CI* confidence interval, *HR* hazard ratio, *SD* standard deviation

**Table 2:** Interaction effect between 1-month match exposures (per 2-SD [3.2 FGE] change) and 12-month match exposure quartiles

12-month match exposure quartiles	HR for effect of 1-month match exposure (90% CI)	*p* value	Inference	% likelihood effect is beneficial|trivial|harmful
<12 (reference)	1.00			
12–21	1.05 (0.88–1.26)	0.64	Unclear	8 |61| 31
22–28	0.92 (0.75–1.12)	0.47	Unclear	44 |50| 6
>28	0.78 (0.63–0.98)	0.07	Likely beneficial	85 |15| 0

*CI* confidence interval, *FGE* full-game equivalent, *HR* hazard ratio, *SD* standard deviation

Modified Wald tests for the within-team and within-player random effects were both significant (*p* ≤ 0.05) and therefore provided evidence of correlation between observations from the same team as well as between recurrent events within individual players. Table 3 displays the model selection criteria for the three survival models. The Cox PH model had the poorest fit on all three selection criteria. The nested frailty model performed significantly better than both the Cox PH model and the shared frailty model.

**Table 3:** Model selection criteria for the three fitted survival models

Model	Model selection criteria		
	LL	AIC	BIC
Cox PH	−36,809	264	267
Shared frailty	−36,685^a^	125^a^	91^a^
Nested frailty	−36,682^a,b^	120^a,b^	88^a,b^

*AIC* Akaike information criterion, *BIC* Bayesian information criterion, *LL* log likelihood, *PH* proportional hazards

^a^Substantial improvement compared with Cox PH model fit

^b^Substantial improvement compared with shared frailty model fit

## Discussion

This is the first study to specifically investigate match exposures as a risk factor for injury in professional rugby union players. It is also the first application of the nested frailty model for the analysis of recurrent injury events in a multi-team setting. The results demonstrate that 12-month match exposure is associated with injury risk in a non-linear fashion; players involved in fewer than ≈15 or more than ≈35 matches over the preceding 12-month period were at an increased risk of injury. Monthly match exposure was linearly and positively associated with injury risk, such that a higher recent load increased injury risk, although this effect was attenuated for players in the upper quartile for 12-month match exposures (>28 matches).

The 12-month match exposure variable displayed a substantial association with current injury risk. A cubic relationship was evident, with heightened injury risk evident for players who played fewer than ≈15 or more than ≈35 matches in the preceding 12 months. In qualitative investigations, professional rugby union players have attributed factors such as limited recovery time in the off season and an ‘anti-rest culture’ as causes for burnout syndrome and increased injury incidence [8]. The results of the current study concur with these findings and provide the first quantitative evidence of an increased injury risk when players are involved in an exceptionally high number of matches in the preceding 12 months. High match exposure demands in the preceding 12-month period may result in cumulative fatigue, reducing the stress-bearing capacity of tissue and thus increasing the likelihood of injury [36]. Fatigue effects incurred cumulatively may also alter neuromuscular control responses, such that potentially hazardous movement strategies are employed that increase the likelihood of injury [37]. In addition, the psychological [38], travel [39], and training [12] demands associated with involvement in a high number of professional rugby union matches are also likely to contribute to the observed injury risk. The total number of players involved in more than 35 matches over the course of a 12-month period was relatively small (*n* = 79) and likely represents an elite (international-level) sub-group of players [23]. Limiting this group’s 12-month match exposures to involvement in 35 matches should be considered as a route to reducing their injury risk. Currently, members of England’s elite player squad are restricted to playing a maximum of 32 FGE per season [40]. The current study considered match involvements, rather than FGE, to account for the training, psychological, and travel loads associated with each match involvement [23]. However, a supplementary analysis performed using the number of FGE in the preceding 12 months (see the Electronic Supplementary Material) demonstrated a similar increase in risk for high match exposure values (at >30 FGE) and thus provided additional evidence for avoiding exceptionally high match exposure levels.

The observed non-linear relationship, with a reduction in injury risk between 12-month match exposures of 15–35 matches, may be indicative of the protective effects of acquiring an appropriate level of match-specific fitness and physical robustness [12]. A similar ‘U-shaped’ relationship has been observed between 4-week cumulative training loads and injury risk in this population [41]. To alleviate their risk of injury, players involved in a low (fewer than ≈15) number of matches over the preceding 12-month period may benefit from additional match-intensity conditioning sessions or match exposures at lower playing levels, whereas players involved in a high number of matches (more than ≈35) may benefit from careful monitoring and potentially modified training/match exposures, longer off-season rest periods, and/or bespoke recovery/prehabilitation measures.

The number of matches played in the preceding 30-day period (1-month match exposure) was linearly and positively associated with current injury risk. Evidence from professional football populations suggests that congested fixture periods can lead to fatigue and an increased risk of injury in the ensuing period [10, 11]. The direct physical contact between players during rugby union matches, in combination with the high physiological demands [42] associated with its high-intensity, intermittent nature [43], prolongs the time-course to full physiological recovery following a match in comparison with football [44] and thus fixtures are typically separated by at least 6 days. The results of the present study indicate that accumulating match exposure over a 30-day period increases a player’s current injury risk in a linear fashion, although the impact of 1-month match exposure was attenuated for players in the upper quartile of 12-month match exposures (>28 matches). This moderation effect implies that players who have accumulated high match exposures over the past 12 months are better able to cope with high monthly match exposures. This finding is analogous to recent work describing the acute: chronic workload with respect to daily training loads and the importance of considering the loads for which players have been prepared [12]. Here, the ‘acute’ and ‘chronic’ timeframes differed from the usual 1-week and 4-week periods [45], respectively, because of the nature of match exposure (i.e., typically one fixture per week) and to help inform pertinent questions relating to fixture scheduling and match workload limits [23]. The influence of different between-match recovery times and multiple consecutive fixtures on injury rates warrants investigation in future studies.

The present study provides novel evidence for both within-team and within-player clustering of injury survival times in elite rugby union players. The within-player clustering confirms that injury survival times are correlated via a common risk factor or injury mechanism to which the individual is exposed (e.g., a genetic predisposition to ligament injuries) [46, 47]. The within-team clustering of observations may be indicative of the injury risk associated with a given team’s training and match practices (e.g., aggressive defensive tactics), the nature of their injury reporting practices, or both. The frailty model has previously been identified as the most appropriate survival model for sports injury recurrent events [16], but this is the first study to consider both within-player and within-team clustering. The present study confirms the importance of accounting for clustering effects in sport medicine research [48] and demonstrates the utility of the nested frailty model for survival analyses with more than one level of clustering.

In agreement with the majority of current research [49–51], past injuries were shown to influence a player’s subsequent injury risk (after adjustment for age), although this is the first study to investigate this relationship amongst professional rugby union players. Notably, including the player as a random effect variable within our statistical model prevented the bias away from the null associated with typical analyses of this risk factor [52] and thus provided robust evidence for previous injury as a causal risk factor for subsequent injury. Following an injury, alterations to a player’s intrinsic risk factors may occur (e.g., altered movement patterns, loss of balance, or other psychological/functional impairments), which may modify the player’s future predisposition to injury [53, 54]. It may be that modified recovery and rehabilitation strategies are required for players with substantial previous injury histories to help reduce the injury burden associated with this risk factor. All other covariates included in the nested frailty model (age, mass, height, and positional group) had trivial effects on overall injury risk, implying that these factors have minimal influence on injury risk compared with the effects of match loads and previous injury history.

A limitation of the current study is the absence of an ‘intensity’ measure for the match exposures undertaken by players. Whilst between-player variation in subjective ratings of effort (ratings of perceived exertion [RPE]) for rugby union matches has been reported to be trivial [55], external load measures (e.g., number of collisions or PlayerLoad™) may be helpful in quantifying the overall load placed on players during matches and thus could improve the sensitivity of the match exposure variable with regards to injury risk. However, such external load measures require further validation [56], particularly with respect to quantifying the contact loads inherent to rugby union [57]. External load measures were not available in this large multi-team study. Similarly, individual training loads were not accounted for in the current study. Training loads are likely to moderate the relationship between match exposures and injury [58] and so an integrative multi-team study that considers both individual match and individual training loads, alongside other key risk factors such as previous injury history [50, 59] and psychological stress [38], is required to fully understand the pathway between player workloads and injury.

## Conclusion

This study demonstrates that players who have been exposed to low (<15) or exceptionally high (>35) 12-month match exposures have a substantially higher current injury risk. Month match exposures are linearly and positively associated with injury risk, although this effect is attenuated for players in the upper quartile for 12-month match exposures (>28 matches). These data make a major contribution to the support decisions relating to player workload management at individual clubs as well as to decisions regarding fixture scheduling and policies relating to player match exposure limits for sport administrators.

## Electronic supplementary material

Below is the link to the electronic supplementary material.

Supplementary material 1 (PDF 10 kb)
