# Supplementary material for: How Much Rugby is Too Much? A Seven-Season Prospective Cohort Study of Match Exposure and Injury Risk in Professional Rugby Union Players
Source: Sports Med. 2017 Mar 30;47(11):2395–402. doi: 10.1007/s40279-017-0721-3 (PMC5633632; doi:10.1007/s40279-017-0721-3)
Supplement: Supplementary file 1 — Supplementary material 1 (PDF 10 kb) [file 40279_2017_721_MOESM1_ESM.pdf]

Supplementary Material

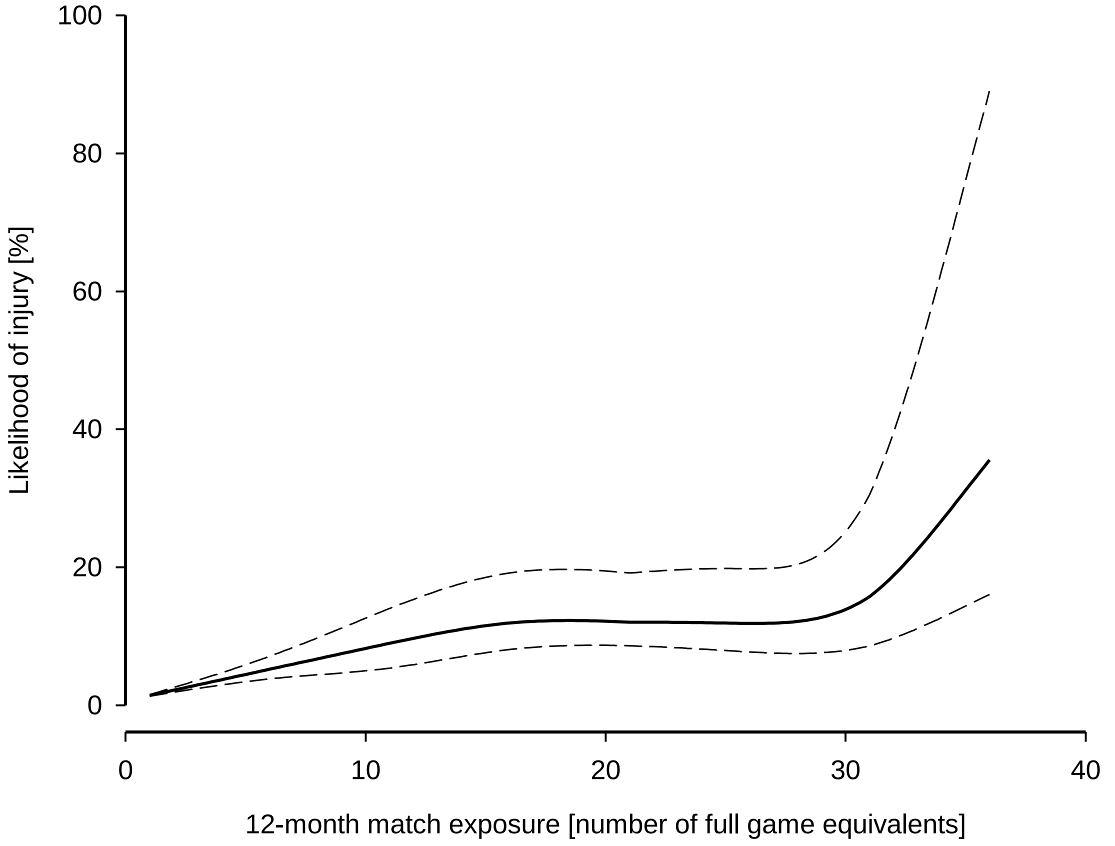

Figure S1. Non-linear association between injury risk and 12-month match exposure, calculated using full-game equivalents rather than number of match involvements.
